# Estimation of Dietary Exposure to Sodium Benzoate (E211) and Potassium Sorbate (E202) of Children and Adolescents in the Oran Region, Algeria

**DOI:** 10.3390/foods13233880

**Published:** 2024-11-30

**Authors:** Fatima Saida Zouaoui, Rachid Boukhari, Nabila Heroual, Nadjette Djemouai, Dalal Redouane, Djamel Saidi, Samia Addou, Omar Kheroua

**Affiliations:** 1 Laboratory of Physiology of Nutrition and Food Safety, Department of Biology, Faculty of Natural and Life Sciences, University Oran 1 Ahmed Ben Bella, Oran 31000, Algeria; s.zouaoui@esa-mosta.dz (F.S.Z.);; 2 Higher School of Agronomy, Mostaganem 27000, Algeria; 3 Laboratory of Biotechnology Applied to Agriculture and Environmental Preservation, Mostaganem 27000, Algeria; 4 Public Establishment Specializing in Pediatric Oncology, Oran 31000, Algeria; 5 Department of Biology, Faculty of Natural and Life Sciences and Earth Sciences, University of Ghardaia, Ghardaïa 47000, Algeria; 6 Microbial Systems Biology Laboratory, Kouba Higher Normal School, Algiers 16000, Algeria; 7 Higher School of Biological Sciences of Oran, Oran 31000, Algeria

**Keywords:** sodium benzoate, potassium sorbate, food exposure, HPLC

## Abstract

Sodium benzoate (E211) and potassium sorbate (E202) are two preservatives widely used alone or in combination in the food industry in Algeria. This study aims to estimate the dietary exposure of children and adolescents to these substances in Oran Province (Algeria) and to assess the risks incurred in consuming these two preservatives. For this, a total of 116 commercial food samples were analyzed for potassium sorbate and sodium benzoate content by HPLC, and a survey was carried out on 250 children and 765 adolescents using a consumption frequency questionnaire as the method. The concentration of preservatives in foodstuffs was mostly within the maximum permitted limits set by national and international standards. In scenario 2 and scenario 3, the estimated average dietary exposure to the two preservatives was below the ADI: it was 17–48% and 21–36% of the ADI for potassium sorbate and sodium benzoate, respectively. The dietary exposure at the 95th percentile exceeded the ADI for sodium benzoate and potassium sorbate when calculations were made using the average of samples containing these preservatives. The main contributors to exposure were carbonated drinks for sodium benzoate and juices, cheeses, and yogurts for potassium sorbate.

## 1. Introduction

Preservatives are used in foods mainly to inhibit the growth of bacteria, molds, fungi, and yeasts [1]. Among these preservatives, sodium benzoate (E211) and potassium sorbate (E202) are the most commonly used in the food industry [2,3]. However, the presence of these preservatives at levels above the maximum permitted limits can be harmful to human health [4]. Several studies have shown that increased potassium sorbate intake can cause cytotoxic and genotoxic effects by producing mutagenic compounds and inducing chromosomal aberrations. These effects can result in the development of numerous chronic diseases, including diabetes mellitus, cancer, etc. [5]. Sodium benzoate can be converted by de-carboxylation into toxic benzene, particularly in combination with ascorbic acid, and then becomes a highly toxic, mutagenic, teratogenic, and carcinogenic compound [4]. Furthermore, research indicates that sodium benzoate induces oxidative stress and detrimentally affects the immune system, liver, kidneys, fertility, neurotransmission, and cognitive function [4,6].

The maximum permitted concentrations of preservatives in each type of food are specified in the Algerian Food Code published in 2012 by the Algerian Ministry of Commerce and also in the International Food Code adopted in 1995 by the Food and Agriculture Organization (FAO) and the World Health Organization (WHO), which was recently revised in 2023. Moreover, the Acceptable Daily Intake (ADI) is the amount of a chemical that can be consumed daily without causing any adverse effects or harm to a person’s health during their entire lifetime [7]. Several health organizations have established the ADI of sodium benzoate and potassium sorbate; it is set at 5 mg/kg b.w./d for sodium benzoate and 11 mg/kg b.w./d for potassium sorbate by the European Food Safety Authority (EFSA) [8,9].

The estimation of dietary exposure is based on data on levels of the substance in food and consumption data for the foods concerned. This estimation can be conducted using the deterministic, refined deterministic, or probabilistic approaches [10]. The deterministic approach consists of using a single value for the concentration of the substance in the food and food consumption to estimate the dietary exposure of a population. This approach fails to reflect the real situation of the population since it assumes that all individuals consume the same quantity of a specific food with a fixed additive concentration. The refined deterministic approach consists of using an empirical distribution of food consumption combined with a single value for the concentration of the substance. This approach has the advantage of being more informative than the deterministic approach since it takes into account the variability of food consumption between individuals in the population studied. However, it has the disadvantage of being conservative since it assumes that a specific food always contains the same danger and is always present at the same level. The probabilistic or stochastic approach consists of generating a distribution of dietary exposures using an empirical distribution of food consumption combined with a distribution of the chemical concentration. This approach requires more detailed data, which enables a more accurate estimate of consumer exposure [10,11].

Several studies assessing dietary exposure to preservatives have been conducted worldwide. The Joint FAO/WHO Expert Committee on Food Additives (JECFA) [12] and the European Food Safety Authority (EFSA) [13] studied dietary exposure to benzoates and sorbates, respectively. The average exposure in these studies did not surpass the Acceptable Daily Intake (ADI) within the population under investigation. However, consumers at higher percentiles exceeded the ADI for some age groups [12,13]. The results of dietary exposure studies cannot be extrapolated, as food behavior and manufacturing practices vary significantly from one country to another and from one region to another [14]. Therefore, due to the absence of available data in Algeria regarding the levels of food preservatives and the consumption of preservatives in food, we thought it would be useful to carry out a study on the exposure of children and adolescents to two commonly used preservatives in the food industry. These groups are at a high risk of surpassing the ADI for food additives, as their weight is lower than that of adults.

The objectives of the present study are (1) to determine the concentration of potassium sorbate and sodium benzoate in foods available on the Oran market, (2) to evaluate the dietary exposure to potassium sorbate and sodium benzoate of children and adolescents living in the Oran region and assess the risk posed by these preservatives, and (3) to identify the main foods that contribute to food exposure among consumers.

## 2. Materials and Methods

### 2.1. Dosage of Potassium Sorbate and Sodium Benzoate

#### 2.1.1. Selection of the Studied Food Products

A survey was undertaken to establish a fairly exhaustive list of food products likely to contain these preservatives. This survey was based on the lists of ingredients displayed on the packaging of products on the Oran market. A total of 116 samples were analyzed, of which 70 were used to determine potassium sorbate (E202) content and 46 to determine sodium benzoate (E211) content. These studied samples were grouped into 12 food types: 5 for sodium benzoate: carbonated drinks, juices, energy drinks, flower waters, flavors, and mayonnaise; and 9 for potassium sorbate: juices, dairy desserts, cheeses, margarine and butter, cakes and madeleines, bricks, mayonnaise, ketchup, and candy. Each sample was analyzed twice.

#### 2.1.2. HPLC Analyses

Analyses were performed on an Agilent 1100 series HPLC equipped with a C-18, 5 μm column consisting of a quaternary pump (G1311A), an autosampler (G1313A), a vacuum degasser (G1379A), a column compartment (G1316A), and a diode array detector (G1315B). The mobile phase consisted of a mixture of methanol/phosphate buffer (15/85) (*v*/*v*) delivered at a flow rate of 1 mL/min. The injection volume was set at 10 μL, and detection was performed at 235 nm for sodium benzoate and 254 nm for potassium sorbate. Extraction was performed with methanol/bidistilled water (2/1) (*v*/*v*).

For the calibration curve, a stock solution was prepared by dissolving 20 mg of sodium benzoate or potassium sorbate in 100 mL of bidistilled water (200 ppm). Serial dilutions were performed to obtain calibration solutions with concentrations of 10, 20, 30, 50, 100, and 200 mg/L for sodium benzoate and 5, 10, 20, 30, 100, and 200 mg/L for potassium sorbate. The linear calibration curves obtained follow the subsequent functions:


$$\mathrm{Sodium}\;\mathrm{benzoate}:\;y=2.9*x+0.87\;(R_{2}=0.99987)$$

$$\mathrm{Potassium}\;\mathrm{sorbate}:\;y=53.02*x+64.63\;(R_{2}=0.99981)$$

y corresponds to the chromatographic peak area.

x corresponds to the additive concentration expressed in mg/L.

### 2.2. Food Survey

#### 2.2.1. Target Population and Sampling

The target population was children (6 to 10 years old) and adolescents (11 to 18 years old, subdivided into two age groups: 11 to 14 years old and 15 to 18 years old) attending school in the Oran region of Algeria. The inclusion criteria were age, correct and complete completion of the dietary questionnaire, and absence of any disease that might affect usual food consumption in terms of quality and quantity. Oran Province comprises 26 municipalities, representing a total of 1,453,152 inhabitants, of which 374,991 individuals are aged between 5 and 19. The sampling method used in this study consisted of drawing lots to select a representative sample of the Oran population belonging to the target age group. This sample was randomly fixed at 1000 individuals. First, Oran Province was divided into four strata according to the number of inhabitants per municipality. Then, within each stratum and for each age group, two successive draws were made: the first to select schools and the second to select classes from the list of schools previously drawn. The draws were carried out using Epi Info 7 software.

#### 2.2.2. Anthropometric Measurements

Weight measurements were carried out on-site on the day of the survey using a digital scale calibrated with a precision of 0.1 kg. Then, the values obtained were recorded on each participant’s questionnaire.

#### 2.2.3. Living Standards and Socio-Demographic Data

The living standards of each subject surveyed were assessed according to monthly income and the number of children in the household, with three categories of classification: low, medium, and high. Additional information on parents’ educational level and profession was also collected. The parents’ professional activity was classified according to the National Statistics Office socio-professional nomenclature. This part of the questionnaire was completed by the pupils’ parents.

#### 2.2.4. Food Consumption Data

This study was carried out in 2013–2014 with a frequency-of-consumption questionnaire specially developed for this survey to obtain accurate information on the quantity consumed and the frequency of consumption of the various marketed foods containing the targeted preservatives. The questionnaire listed eight foods grouped into five categories: (1) non-alcoholic beverages, (2) dairy products, (3) fats, oils, and fat emulsions, (4) sauces and similar products, and (5) confectionery. The frequency of consumption was divided into two categories: “yes every day” at one of four levels (one, two, three, or four times a day); or “no”, which included four levels too (one, two, or three times a week, or never). A preliminary version of the prepared questionnaire was tested on around twenty people to assess its comprehensibility. The prepared questionnaire also allowed students to note the brands of foods and beverages consumed and their quantities in household units. The amount of food consumed was accurately reported according to the type of food, thanks to a wide choice of options for the subjects surveyed. These portions were presented during the investigation as a photo album. The weight of the food portions mentioned in the questionnaire, expressed in grams, was determined in advance in the Laboratory of Physiology of Nutrition and Food Safety of the University of Oran 1 Ahmed Ben Bella.

### 2.3. Data Processing

#### 2.3.1. Estimation of Dietary Exposure

Estimated dietary exposure to preservatives was obtained for each subject by multiplying the average daily intake of each type of food by the concentration of the preservative present in that food, then, for each preservative, adding up the intakes from all dietary sources. Finally, this intake (mg/day) was divided by the actual body weight measured for each participant (kg), so that it could be expressed in mg/kg b.w./day [10]. The calculation results are presented as the mean and 95th percentile. Three different scenarios were established to determine dietary exposure to the two preservatives for each subject [15]:

First scenario: The estimate of food exposure was obtained by multiplying the individual food data collected by the maximum permitted concentrations.

Second scenario: The estimate of food exposure was obtained by multiplying the individual food data collected by the average of the real concentrations of only the samples containing the preservative, assuming that the consumer is faithful to the brand and continues to consume food products that contain the targeted preservative.

Third scenario: The estimate of food exposure was obtained by multiplying the individual food data collected by the average of the real preservative concentrations of all samples for each food type (including zero values), assuming that the consumer is not brand-loyal and consumes food products randomly, containing or not containing the target preservatives.

#### 2.3.2. Risk Assessment

Risk is calculated from food exposure and ADI according to the following formula:

Risk(%)=(Foodexposure/ADI)∗100

The calculated risk must be less than 100% to indicate that no risk would result from dietary exposure to the preservative over a consumer’s lifetime. The ADI values for sodium benzoate and potassium sorbate used in this study were 5 mg/kg b.w./d and 11 mg/kg b.w./d, respectively.

### 2.4. Statistical Analysis

All statistical analyses in this study were carried out using SPSS (Statistical Package for the Social Sciences) software version 22. Student’s *t*-test was used to examine significant differences between the two sexes. Significant differences between age groups and living standards were examined using one-way ANOVA and the Duncan test.

## 3. Results

### 3.1. Characteristics of the Studied Population

This study evaluated the dietary exposure to sodium benzoate (E211) and potassium sorbate (E202) of 1015 pupils (250 children and 765 adolescents). The participation rate was 93.46%: 39 pupils refused to participate, 11 were absent on the day of the survey, and 21 responses were eliminated. The distribution of the final sample by strata and age groups is shown in Supplementary Table S1.

The participants had an average body weight of 28.40 ± 0.42 kg, 48.07 ± 0.67 kg, and 59.27 ± 0.54 kg for the age groups 6–10 years, 11–14 years, and 15–18 years, respectively. Furthermore, additional socio-demographic information on the presence of parents, as well as their level of education and professional situation, for the three living standards considered are described in Supplementary Table S2.

### 3.2. Concentration of Preservatives in Foods

The sodium benzoate and potassium sorbate concentrations in the foods analyzed are presented in Table 1. Sodium benzoate was detected in 63.04% of the products analyzed; the highest average concentration was recorded in mayonnaise with a value of 205.12 ± 7.43 mg/kg, and the lowest was obtained in energy drinks with a value of 54.37 ± 3.32 mg/kg. The average concentration of sodium benzoate in carbonated drinks was 148.09 ± 22.92 mg/kg, while that of juices was 90.95 ± 5.46 mg/kg. None of the samples exceeded national or GSFA standards.

Potassium sorbate was detected in 65.71% of the products analyzed, with the highest average concentration in melted cheese with a value of 1667.24 ± 129.43 mg/kg, and the lowest was obtained in dairy desserts with a value of 209.36 ± 21.34 mg/kg. In juices, margarine, and candies, the average concentration of potassium sorbate was 279.62 ± 76.32 mg/kg, 621.10 ± 61.99 mg/kg, and 467.01 ± 29.21 mg/kg, respectively. Potassium sorbate was also detected in bakery products (madeleines and cakes) at 994.44 ± 177.1 mg/kg and bricks at 577.33 ± 31.2 mg/kg. The average potassium sorbate concentration in mayonnaise was 966.56 ± 261.67 mg/kg, while that in ketchup was 868.13 ± 137.64 mg/kg. For these two types of products, only one sample exceeded the national and GSFA standards.

### 3.3. Dietary Exposure to Preservatives

**Figure 1:**
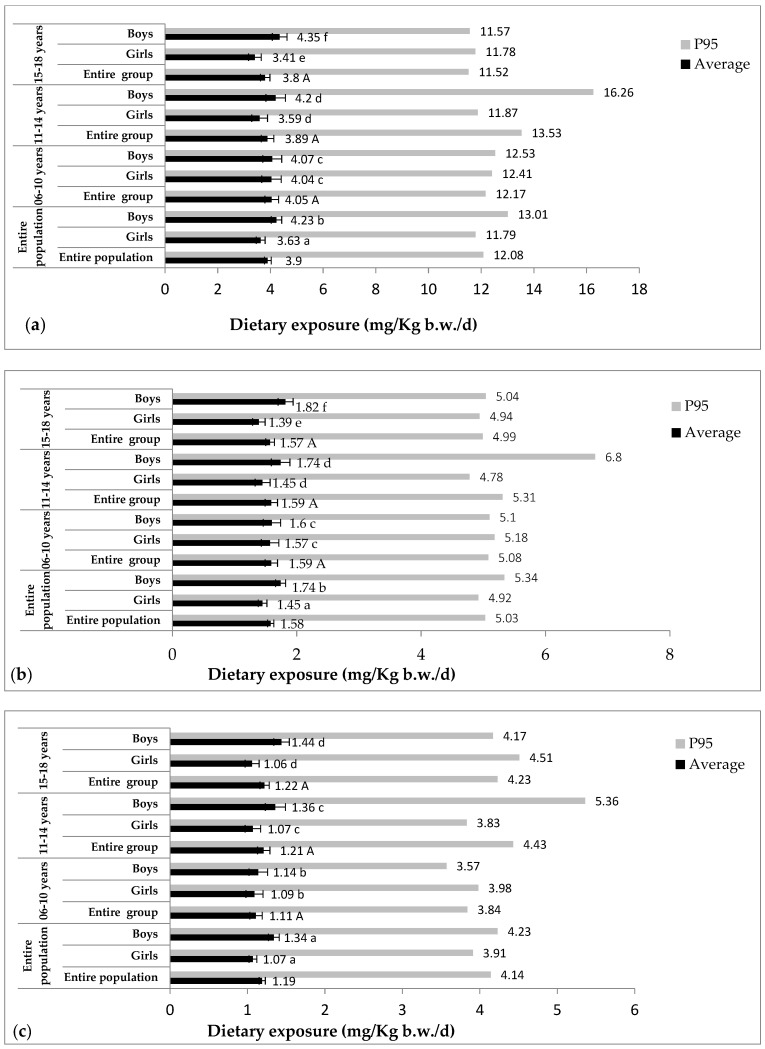
Estimated dietary exposure of consumers (children and adolescents) in the Oran region to sodium benzoate. (**a**): Scenario 1, (**b**): Scenario 2, (**c**): Scenario 3. Same lowercase letters indicate no significant differences between sexes. Same uppercase letters indicate no significant differences between the three age groups. Data are presented as the mean ± SE.

**Figure 2:**
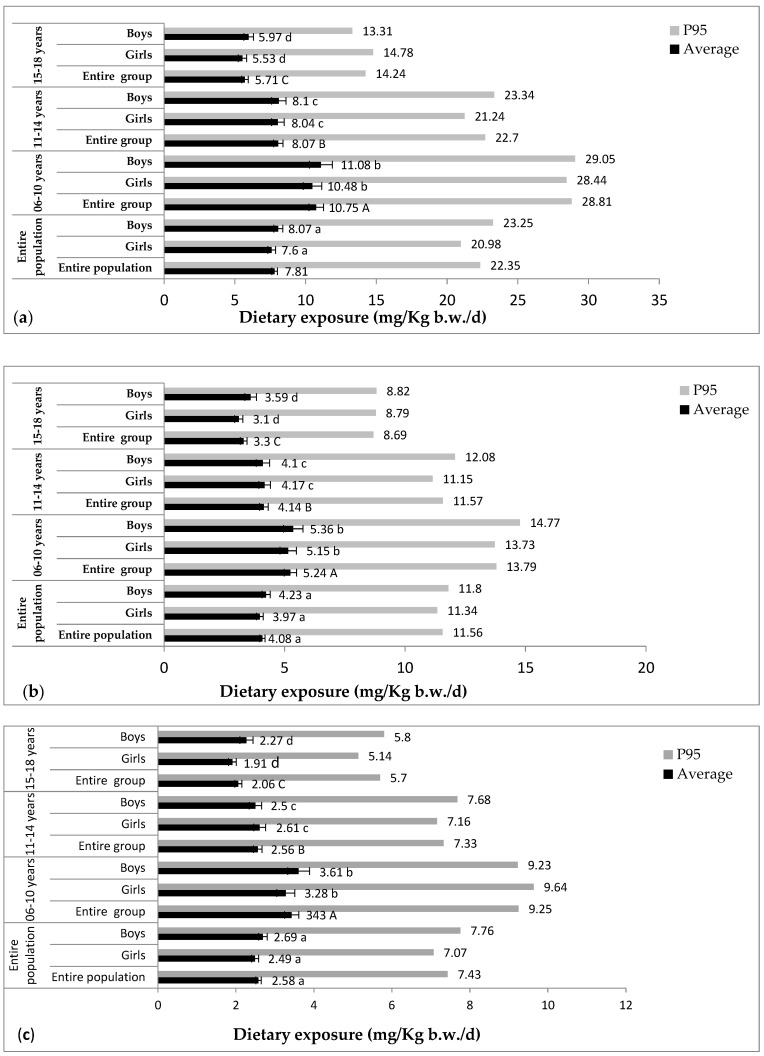
Estimated dietary exposure of consumers (children and adolescents) in the Oran region to potassium sorbate. (**a**): Scenario 1, (**b**): Scenario 2, (**c**): Scenario 3. Same lowercase letters indicate no significant differences between sexes. Same uppercase letters indicate no significant differences between the three age groups. Data are presented as the mean ± SE.

Figure 1 and Figure 2 summarize the results for exposure average and exposure at the 95th percentile for consumers; Supplementary Table S3 shows the exposure average and exposure at the 95th percentile for consumers in relation to living standards; and Table 2 summarizes the main contributors to dietary exposure to preservatives.

#### 3.3.1. Sodium Benzoate

The survey results revealed that 97% of the population surveyed were sodium benzoate consumers.

In the first scenario, the average exposure and exposure at the 95th percentile for the consumer groups are 3.90 ± 0.13 mg/kg b.w./d (78% of the ADI) and 12.08 mg/kg b.w./d (241.6% of the ADI), respectively. No significant differences were observed between age groups (*p* > 0.05). The highest average exposure was observed in children aged 6 to 10 years (4.05 ± 0.26 mg/kg b.w./d (81% of the ADI)), with a 95th percentile exposure exceeding the ADI (12.17 mg/kg b.w./d (243.4% of the ADI)).

The average exposure to sodium benzoate in the second scenario was 1.58 ± 0.05 mg/kg b.w./d (31.6% of the ADI). However, the exposure at the 95th percentile also exceeded the ADI in this scenario (5.03 mg/kg b.w./d, 100.6% of the ADI). No significant difference was observed between age groups (*p* > 0.05) (from 1.57 ± 0.07 (31.4% of the ADI) to 1.59 ± 0.01 mg/kg b.w./d (31.8% of the ADI)), but there was a significant difference (*p* < 0.05) between boys (1.74 ± 0.08 mg/kg b.w./d, 34.8% of the ADI) and girls (1.45 ± 0.07 mg/kg b.w./d, 29% of the ADI).

When calculations were based on the average of all samples (scenario 3), the average exposure and even the 95th percentile exposure for the consumer group were below the ADI (Ave: 1.19 ± 0.04 mg/kg b.w./d (23.8% of the ADI), P95: 4.14 mg/kg b.w./d (82.8% of the ADI)). No significant difference (*p* > 0.05) was observed between the two sexes (girls: 1.07 ± 0.05 mg/kg b.w./d, 21.4% of the ADI; boys: 1.34 ± 0.07 mg/kg b.w./d, 26.8% of the ADI) or between age groups (from 1.11 ± 0.08 (22.2% of the ADI) to 1.22 ± 0.08 mg/kg b.w./d (24.4% of the ADI)).

In the first scenario, around 26% of sodium benzoate users exceeded the ADI of 5.00 mg/kg b.w./d. By contrast, in the second and third scenarios, the percentage of consumers exceeding the ADI was lower: 5% and 3%, respectively. Carbonated drinks were the main contributor to exposure to sodium benzoate in all three scenarios.

The difference between the three levels was highly significant in relation to living standards (*p* < 0.001). Children with high standards of living were most exposed to sodium benzoate: their average exposure (scenario 2: 2.98 ± 0.15 mg/kg b.w./d; scenario 3: 2.73 ± 0.18 mg/kg b.w./d) was four times higher than that of low-living-standard children (scenario 2: 0.74 ± 0.03 mg/kg b.w./d; scenario 3: 0.64 ± 0.03 mg/kg b.w./d) and twice that of medium-living-standard children (scenario 2. 1.53 ± 0.07 mg/kg b.w./d, scenario 3. 1.17 ± 0.05 mg/kg b.w./d). The highest P95 was in the high-living-standard group with a value exceeding the ADI (scenario 2: 7.96 mg/kg b.w./d (159.2% of the ADI); scenario 3: 7.37 mg/kg b.w./d (147.4% of the ADI)). In contrast, the lowest class had the lowest P95, which did not exceed the ADI (scenario 2: 2.12 mg/kg b.w./d (42.4% of the ADI); scenario 3: 2.22 mg/kg b.w./d (44.4% of the ADI)).

#### 3.3.2. Potassium Sorbate

The survey results show that almost all the pupils involved (99.80%) were consumers of potassium sorbate.

In the first scenario, average exposure and 95th percentile exposure for the consumer group were 7.81 ± 0.20 mg/kg b.w./d (71% of the ADI) and 22.35 mg/kg b.w./d (203.18% of the ADI), respectively.

The difference between age groups was highly significant (*p* < 0.001). The highest average and 95th percentile exposures were observed in children aged 6 to 10 years (Ave: 10.75 ± 0.51 mg/kg b.w./d (97.73% of the ADI); P95: 28.81 mg/kg b.w./d (261.91% of the ADI)).

In this first scenario, P95 exceeded the ADI in all age categories studied, ranging from 121% to 264.09%.

In the other two scenarios, the average exposure for the consumer group was also below the ADI (scenario 2: Ave: 4.08 ± 0.11 mg/kg b.w./d (37.09% of the ADI); scenario 3: Ave: 2.58 ± 0.07 mg/kg b.w./d (23.45% of the ADI)). However, exposure at the 95th percentile exceeded the ADI in scenario 2 (P95: 11.56 mg/kg b.w./d (105.09% of the ADI)) and was below the ADI in scenario 3 (P95: 7.43 mg/kg b.w./d (67.55% of the ADI)).

No significant difference was observed between girls (scenario 2: 3.97 ± 0.14 mg/kg b.w./d (36.09% of the ADI); scenario 3: 2.49 ± 0.09 mg/kg b.w./d (22.64% of the ADI)) and boys (scenario 2: 4.23 ± 0.17 mg/kg b.w./d (38.45% of the ADI); scenario 3: 2.69 ± 0.11 mg/kg b.w./d (24.45% of the ADI)) (*p* > 0.05). However, the difference was very highly significant between age groups (*p* < 0.001). The highest average exposure and 95th percentile exposures were observed in children aged from 6 to 10 years (scenario 2: Ave: 5.24 ± 0.26 mg/kg b.w./d (47.64% of the ADI); P95: 13.79 mg/kg b.w./d (125.36% of the ADI); scenario 3: Ave: 3.43 ± 0.18 mg/kg b.w./d (31.18% of the ADI); P95: 9.25 mg/kg b.w./d (84.09% of the ADI)), followed by children aged from 11 to 14 (scenario 2: Ave: 4.14 ± 0.18 mg/kg b.w./d (37.64% of the ADI); P95: 11.57 mg/kg b.w./d (105.18% of the ADI); scenario 3: Ave: 2.56 ± 0.11 mg/kg b.w./d (23.27% of the ADI); P95: 7.33 mg/kg b.w./d (66.64% of the ADI)). Lastly, the least exposed subjects were aged between 15 and 18 years (scenario 2: Ave: 3.30 ± 0.14 mg/kg b.w./d (30% of the ADI); P95: 8.69 mg/kg b.w./d (79% of the ADI); scenario 3: Ave: 2.06 ± 0.09 mg/kg b.w./d (18.73% of the ADI); P95: 5.70 mg/kg b.w./d (51.82% of the ADI)).

In the first scenario, 20.63% of potassium sorbate consumers exceeded the ADI of 11.00 mg/kg b.w./d. In contrast, in the second and third scenarios, the percentage of consumers exceeding the ADI was lower: 5.63% and 1.09%, respectively. The main contributors to potassium sorbate exposure were juices, yogurts, and cheeses.

Concerning living standards, a very large significant difference was observed between the three living standards (*p* < 0.001); namely, the mean and 95th percentile exposures of children in the high standard of living group (scenario 2: Ave: 6.90 ± 0.29 mg/kg b.w./d; P95: 15.49 mg/kg b.w./d; scenario 3: Ave: 3.97 ± 0.18 mg/kg b.w./d; P95: 10.02 mg/kg b.w./d) were three times higher than those of the children in the low standard of living group (scenario 2: Ave: 2.24 ± 0.07 mg/kg b.w./d; P95: 5.33 mg/kg b.w./d; scenario 3: Ave: 1.51 ± 0.06 mg/kg b.w./d; P95: 4.12 mg/kg b.w./d). In addition, the values obtained for children in the medium standard of living group (scenario 2: Ave: 4.3 ± 0.17 mg/kg b.w./d; P95: 9.83 mg/kg b.w./d; scenario 3: Ave: 2.85 ± 0.12 mg/kg b.w./d; P95: 7.26 mg/kg b.w./d) were almost two times higher than those of the children in the low standard of living group.

## 4. Discussion

Sodium benzoate (E211) and potassium sorbate (E202) are commonly employed as preservatives, either individually or in combination, in the food industry to prolong the lifespan of food products and inhibit the growth of microorganisms [2,16,17]. These preservatives are generally considered safe for most consumers as long as their concentration levels in food do not surpass the maximum limits permitted by regulations [17] and as long as customers do not consume more than the ADI defined by health organizations. Consequently, under certain conditions of use, chemical preservatives can present potential risks of varying degrees of significance to consumer health.

In the present study, first, the average concentrations of two food preservatives (sodium benzoate and potassium sorbate) were determined in marketed foods; then, dietary exposure to these preservatives was assessed in a sample of children and adolescents using three distinct exposure scenarios.

### 4.1. Concentration of Preservatives in Foods

#### 4.1.1. Sodium Benzoate

Table 3 summarizes the data on concentrations of sodium benzoate in marketed foods according to the literature. The mean concentration of sodium benzoate found in carbonated drinks in this study is close to the average concentrations found in various comparable studies conducted in Belgium (128.3 mg/kg) [18], Portugal (148 mg/kg) [19], Lebanon (120 mg/kg) [14], and Australia (119 mg/kg) [20]. However, the result obtained in the present study is higher than that found in other studies carried out in France (71.2 mg/kg) [21], New Zealand (63.1 mg/kg) [22], Austria (69.8 mg/kg) [23], and Iran (61.75 mg/kg) [16]. In contrast, some studies, such as the one conducted in Brazil [24], reported higher average concentrations (259 mg/kg). Two recent studies conducted in Africa examined the levels of sodium benzoate in carbonated drinks: the first study found values ranging from 5.1 to 277 mg/L in Ghana, while the second found concentrations ranging from 14.25 to 218.32 mg/L in Nigeria [25,26]. Furthermore, sodium benzoate was found in carbonated and energy drinks in Bangladesh, with average concentrations ranging from 96.05 to 285.51 mg/L in carbonated drinks and from 52.92 to 1575.37 mg/L in energy drinks [27,28]. The results are consistent with the findings of Das et al. (2024), who confirmed that benzoate and sorbate are employed as preservatives in carbonated drinks and related beverages in the Bangladeshi market **[17]**. Additionally, they noted that benzoate is the most widely utilized preservative. This was also revealed in our study, where sodium benzoate was found to be the only preservative used in marketed carbonated and energy drinks.

The mean sodium benzoate concentration in juices found in this study is significantly lower than that found in Brazil (495 mg/kg) [24], Austria (210.7 mg/kg) [23], Lebanon (245 mg/kg) [14], and Iran (213.2 mg/kg) [2]. A study conducted in Iran found that juices had lower average values, ranging from 12.23 to 56.80 mg/kg [29]. In Belgium, Vandevijvere et al. (2009) found an average concentration of 128.3 mg/kg in lemonade [18]. In Nigeria, average concentrations of sodium benzoate obtained in marketed juices ranged from 25.80 to 245.10 mg/L [26].

The average concentration of sodium benzoate in mayonnaise found in this study is similar to that found in Iran (243.42 mg/kg) [16] and lower than that found in Belgium (585.6 mg/kg), according to the study carried out by Vandevijvere et al. (2009) [18]. A recent study conducted in the Iranian city of Ourmia examined different types of sauce, specifically mayonnaise, and found that the concentration of sodium benzoate ranged from 10.3 to 673 mg/kg [3].

#### 4.1.2. Potassium Sorbate

Table 4 summarizes the data on concentrations of potassium sorbate in marketed foods according to the literature. The average concentration of potassium sorbate in melted cheese found in this study is comparable to that observed in Australia (1506 mg/kg) [20]. Additional research indicated lower levels of this preservative in many countries, specifically New Zealand (368 mg/kg) [22], Denmark (213 mg/kg) [15], and Brazil (629 mg/kg) [24].

The level of potassium sorbate in margarine obtained in this study is consistent with the findings of a Brazilian study (532 mg/kg) [24]. Cressey and Jones (2009) [22] reported an average concentration of 414 mg/kg in New Zealand, which is similar to the findings of an Australian study (421 mg/kg) [20].

The mean concentration of potassium sorbate detected in juices in this study is higher than that obtained in Australia (191 mg/kg) [20] and New Zealand (216 mg/kg) [22]. In Iran, the mean concentration of potassium sorbate in juices varied between 113.8 and 233.3 mg/kg [2]. A recent study conducted in Nigeria by Magomya et al. (2020) found that the average concentrations of potassium sorbate in fruit juices varied between 1.36 and 122.50 mg/L [26].

Tfouni and Toledo (2002) reported a mean sorbate value of 183.2 mg/kg for yogurt in Brazil [24], which is in line with the results obtained in the present study. Lower values were found in Iran by Amirpour et al. (2015), where the concentration of potassium sorbate in yogurt was 36.2 mg/kg [2].

The average concentrations of potassium sorbate in mayonnaise and ketchup found in this study are similar to those found in Austria, according to the study by Mischek and Krapfenbauer-Cermak (2012) (1008.8 mg/kg for mayonnaise and 486 mg/kg for ketchup) [23]. In a study conducted in Iran (Ourmia), Yazdanfar et al. (2023) found that the concentrations of potassium sorbate in mayonnaise ranged from 0.005 to 460.3 mg/kg [3]. A study conducted in Turkey found that sorbic acid was detected in one out of five ketchup samples at a value of 227.27 mg/kg and in four out of five mayonnaise samples, with concentrations ranging from 0.00 to 892.46 mg/kg [30].

Concerning cakes, a study conducted in Iran showed that the average concentration of potassium sorbate was 127.93 mg/kg [16], while a separate study conducted in Pakistan revealed that the concentrations of sorbic acid were between 49 and 895 mg/kg [31]. In our study, the average concentration of potassium sorbate detected in cakes and madeleines was clearly higher than those mentioned above.

Our study’s results show that the concentrations of potassium sorbate and sodium benzoate vary not only between different types of food, but also between samples within the same food type. This could make it difficult to compare our findings with those of other countries, but it is reassuring to know that almost all of the analyzed samples were within national and international standards.

### 4.2. Dietary Exposure to Preservatives

#### 4.2.1. Sodium Benzoate

Concerning the estimation of dietary exposure to sodium benzoate, the highest values of average exposure and exposure at the 95th percentile were obtained in the first scenario. These results are significantly higher than those obtained in other studies [32,33,34]. For example, in the Flemish population of Belgium, the mean exposure and exposure at the 95th percentile for adolescents (13–18 years) were 2.1 mg/kg b.w./d and 4.9 mg/kg b.w./d, respectively [33].

These high values observed in our estimate are due to the maximum authorized national concentrations, which differ from one country to another. For example, according to the Algerian Ministry of Commerce, the maximum permitted concentration in the Algerian food industry for non-alcoholic beverages, which are the main contributor to the average daily intake of benzoates, is 300 mg/kg. However, the limit in Belgium is set at 150 mg/kg [33]. The first scenario may result in overestimating exposure due to the calculations being based on maximum permitted concentrations.

In the second scenario, exposure to sodium benzoate at the 95th percentile exceeded the ADI (5.03 mg/kg b.w./d (100.6% of the ADI)). In some similar studies, benzoate exposure at high percentiles also exceeded the ADI in children and adolescents [9,15,20].

In the third scenario, the mean and the 95th percentile exposures for the consumer groups remained below the ADI. Similar findings have been reported by several authors [9,11,14,21,22,34,35]. In a study conducted in four different countries, dietary exposure to benzoates from soft drinks among consumers aged 8 to 17 was 1.232 mg/kg b.w./d in Mexico, 0.811 mg/kg b.w./d in Canada, 1.069 mg/kg b.w./d in Brazil, and 1.032 mg/kg b.w./d in the USA [11].

Lower values were recorded in New Zealand, where the average exposure in consumers aged 5 to 18 years ranged from 0.6 (12% of the ADI) to 0.9 mg/kg b.w./d (18% of the ADI). In addition, the highest 95th percentile was observed in girls aged 16 to 18, with a value of 3.00 mg/kg b.w./d (60% of the ADI) [22]. In the present study, the highest P95 was 5.36 mg/kg b.w./d (107.2% of the ADI) in boys aged 11 to 14.

In our survey, carbonated drinks were the main contributors to sodium benzoate exposure. Carbonated drinks have also been identified as the main contributor in several studies, particularly in children and adolescents [9,11,12,14,15,22,34]. They account for 95.3% of children’s benzoate intake in New Zealand (ages 5 to 15) [22], 58% to 77% of children’s benzoate intake in the UK and Ireland [34], and 80% of children’s benzoate intake in the Brazilian population [24,36].

According to our survey, the percentage of sodium benzoate consumers who exceeded the ADI ranged from 3% to 26%, depending on the type of scenario. In a similar study in Denmark covering all age groups, when calculations were based on the average of all samples, more than 5% of the population had a daily intake that exceeded the ADI. In the high-intake calculation, based on the average of samples containing the preservative, around 10% of men and women had intakes higher than the ADI [15].

#### 4.2.2. Potassium Sorbate

Concerning the estimation of dietary exposure to potassium sorbate, the results obtained for the average and 95th percentile exposures in the first scenario are consistent with those reported by the EFSA [13], who report that the mean exposure of adolescents (10 to 17 years) to sorbate is 7.9 mg/kg b.w./d in France and Germany, 7.2 mg/kg b.w./d in Italy, and between 6.4 and 9.1 mg/kg b.w./d in Spain. In our study, the highest mean and 95th percentile exposure values were observed in children aged 6 to 10 years. Similar values were obtained from the EFSA study [13], where mean exposure in children aged 3 to 9 years was 10.1 mg/kg b.w./d in Greece, 10.9 mg/kg b.w./d in Germany, between 11.1 and 11.4 mg/kg b.w./d in Spain, and 11.2 mg/kg b.w./d in Italy.

The average and the 95th percentile exposures in scenario 3, as well as the average exposure in scenario 2, are below the ADI. Similar studies have also shown that mean and high-percentile exposures are below the ADI for the age group 6 to 18 years [13,15,20,22,37]. In New Zealand (scenario 3), the average exposure of the consumer group of children aged 5 to 18 years ranges from 0.7 to 1.2 mg/kg b.w./d (P95: 2.8 to 4.8 mg/kg b.w./d). The highest values in this age group were observed in children aged 5 to 12 years, where girls had an average exposure of 1.1 mg/kg b.w./d (P95: 3.9 mg/kg b.w./d), while boys had an average exposure of 1.2 mg/kg b.w./d (P95: 5.3 mg/kg b.w./d) [22]. Similarly, in our study (scenario 3), children aged 6 to 10 years also showed the highest values, with a mean exposure of 3.28 ± 0.23 mg/kg b.w./d (P95: 9.64 mg/kg b.w./d) for girls and 3.61 ± 0.28 mg/kg b.w./d (P95: 9.23 mg/kg b.w./d) for boys. These values are significantly higher than those found in a New Zealand study.

Finally, it is important to point out that in scenario 1 and scenario 2, the P95 of the surveyed population exceeded the ADI, representing twice the ADI in the case of scenario 1 (203.18%) and 105.09% of the ADI in the case of scenario 2. In addition, children aged 6–10 recorded the highest P95 (scenario 1: 261.91%; scenario 2: 125.36%), which also exceeded the ADI.

Juices, yogurts, and cheeses were the main contributors to potassium sorbate exposure. In New Zealand, refrigerated orange juice, English pancakes, crumpets, muffins, and margarine were the main contributors to sorbate exposure [22].

Percentages of 5.63% and 1.09% of potassium sorbate consumers exceeded the ADI in scenario 2 and scenario 3, respectively. However, in scenario 1, the percentage of consumers exceeding the ADI was 20.63%. In their study on the Danish population, Leth et al. (2010) [15] found that in the calculation of high intakes, more than 10% of men and more than 5% of women exceeded the ADI. However, the ADI was not surpassed when calculations were based on the average of all samples.

Regarding dietary exposure to the two preservatives in accordance with living standards, it was found that there is a positive correlation between average exposure and standard of living, where the higher the standard of living, the higher the average exposure. Thus, children from the highest standard of living category were the most exposed, with average exposures three and four times higher than those in the lowest standard of living group for potassium sorbate and sodium benzoate, respectively. Indeed, in the case of sodium benzoate and at the high percentile, the ADI was exceeded by children in the highest class (scenario 2: 159.2%; scenario 3: 147.4%). However, in this high class, exposure to potassium sorbate at high percentiles also exceeded the ADI, but only in scenario 2 (140.82%). 

### 4.3. Survey Limitations

In this survey, food consumption data were collected using a single method, which was the consumption frequency questionnaire. However, it is essential to note that this method can lead to an overestimation of the frequency of consuming foods that are rarely consumed and an underestimation of the frequency of consuming foods that the respondent considers to be “unhealthy” or “bad” [38]. Nonetheless, this method is more precise than other strategies used to estimate the average dietary exposure to chemicals that have a high daily variability in dietary intake and for which there are only a few important dietary sources. Food frequency questionnaires can also be used to separate food consumers from non-consumers who declare they never eat the food [10].

Scenario 3 offers a more realistic and precise estimation among the three scenarios employed in this survey. This is because it is based on the averages of all concentrations, including zero values. This model does not consider the influence of brand loyalty, unlike scenario 2, which is based on the average concentrations of only the samples containing the preservative. This second scenario can, therefore, result in an overestimation of consumption, especially as the averages used may represent only a small percentage of the products marketed. This is the case, for example, for juices, where only 27.27% of the samples analyzed contained potassium sorbate, but their contribution to dietary exposure was 40.63% in scenario 2. This contribution rate drops to 17.62% in scenario 3. Moreover, the notion of “brand loyalty” must be treated with great caution because a consumer can also be loyal to a brand that does not contain the targeted preservative [39]. Therefore, it is very important to carefully choose the ideal scenario and model for the survey to obtain a precise and true estimation of dietary exposure while also avoiding any underestimation or overestimation.

Another limitation is that the number of samples analyzed in some food categories was relatively low (energy drinks, flower waters and aromas, cheeses, margarines and butter, cakes and madeleines, bricks, ketchups, and candies). For this reason, consumption could be underestimated or overestimated.

Although this study was conducted in 2013/2014, which can be considered as another limiting factor, the results remain interesting and relevant since the collection of the data on consumption and preservative levels in foods was carried out during the same period. Moreover, no similar study has been conducted in Algeria to date. This means that our survey remains the only reference, and its results can be used and exploited. Similar studies are also interesting and essential to assess changes in Algerian consumer behavior, as well as changes in preservative concentrations in marketed products, especially as the Algerian market has expanded in recent years.

## 5. Conclusions

This study aimed firstly to determine the concentrations of sodium benzoate (E211) and potassium sorbate (E202) in agri-food products available on the Oran market, and secondly to estimate the dietary exposure of pupils (three age groups) to sodium benzoate and potassium sorbate and to evaluate the health risk involved. The average concentrations of preservatives in the foods analyzed were generally similar to or lower than those in other countries, and 98,09% of the concentrations obtained complied with the maximum limits allowed in the Algerian Food Standards Code.

In the three scenarios considered, the estimated average exposure to sodium benzoate for the three age groups studied was well below the ADI, and the risk index did not exceed 87%. The average exposure to potassium sorbate for scenario 2 and scenario 3 was also below the ADI for all age groups where the risk did not exceed 48% of the ADI; however, in scenario 1, the average exposure to potassium sorbate reached the ADI for boys in the 6–10 age group (100.73%). On the other hand, dietary exposure to sodium benzoate and potassium sorbate at the 95th percentile exceeded the ADI, especially in the first and second scenarios, where the risk reached its maximum of 270% for sodium benzoate and 264% for potassium sorbate; this concerns the two age groups most exposed to these preservatives, namely 6–10 years followed by 11–14 years. This is because dietary exposure is inversely proportional to body weight, i.e., the lower the weight, the higher the exposure. We also noted that dietary exposure to the two preservatives is closely linked to the standards of living of the studied population, i.e., the higher the standard of living, the higher the exposure. We also determined the main contributors to the intake of the two preservatives: juices, yogurts, and cheeses for potassium sorbate and carbonated drinks for sodium benzoate.

These findings indicate that the use of sodium benzoate and potassium sorbate as preservatives in the food industry can present a risk to consumers, and special attention should be paid to major consumers for whom the risk of being exposed to a level higher than the toxicological reference value remains possible. This suggests that further, more exhaustive studies are essential to gain a clearer picture of this problem, particularly regarding the population at risk (children and adolescents), especially as, to date, no such study has yet been carried out in Algeria.

## Figures and Tables

**Table 1:** Concentration of conservatives in analyzed foods (mg/kg).

Food Category	Food Type	N	Sodium Benzoate					Potassium Sorbate				
			n	Maximum Concent ^1^	Maximum Concent ^2^	Ave ± SE (Scenario 2)	Ave ± SE (Scenario 3)	N	Maximum Concent1	Maximum Concent2	Ave ± SE (Scenario 2)	Ave ± SE (Scenario 3)
14.1. Non-alcoholic Drinks	Carbonated drinks	16	16/16	1000	300	148.09 ± 22.92	148.09 ± 22.92	-	1000	300	-	-
	Juices	11	3/11	1000	300	90.95 ± 5.46	24.80 ± 3.56	3/11	1000	300	279.62 ± 76.32	76.26 ± 7.25
	Energy drinks	6	3/6	600	300	54.37 ± 3.32	27.18 ± 5.80	-	500	300	-	-
	Flower waters and aromas	6	6/6	1000	300	112.1 ± 4.22	112.1 ± 4.22	-	300	300	-	-
01.0. Dairy products and derivatives	Milky desserts	16	-	300	-	-	-	10/16	1000	-	209.36 ± 21.34	130.85 ± 15.28
	Cheeses	6	-	1000	-	-	-	6/6	3000	2000	1667.24 ± 129.43	1667.24 ± 129.43
02.0. Fats, oils and fat emulsions	Margarines and butters	6	-	1000	-	-	-	4/6	2000	2000	621.10 ± 61.99	414.06 ± 35.65
07.0. Bakery Products	Cakes and madeleines	6	-	1000	-	-	-	6/6	1000	2000	994.44 ± 177.1	994.44 ± 177.1
	Bricks	6	-	-	-	-	-	6/6	-	2000	577.33 ± 31.2	577.33 ± 31.2
12.6. Sauces and similar products	Mayonnaise	7	1/7	1000	1000	205.12 ± 7.43	205.12 ±7.43	7/7	1000	1000	966.56 ± 261.67	966.56 ± 261.67
	Catchups	6	-	1000	-	-	-	3/6	1000	-	868.13 ± 137.64	434.06 ± 90.20
05.0. Confec- tionery	Candies	6	-	1500	-	-	-	1/6	1500	-	467.01 ± 29.21	77.83 ± 10.64

^1^ according to GSFA (mg/kg); ^2^ according to national standards (mg/kg). Ave: average; N: number of samples; n: number of samples containing the preservative. SE: standard error.

**Table 2:** Contribution of foods tested to average dietary exposure to sodium benzoate and potassium sorbate.

Food	% Contribution to Average Exposure to Preservatives (Scenario 2/Scenario 3)	
	Sodium Benzoate	Potassium Sorbate
Carbonated drinks	66.98/88.15	0.00/0.00
Juices	33.01/11.84	40.63/17.62
Energy drinks	0.00/0.00	0.00/0.00
Flower waters and aromas	0.00/0.00	0.00/0.00
Milky desserts (e.g., cream desserts, fruity or flavored yogurts)	0.00/0.00	14.57/14.49
Cheeses	0.00/0.00	27.23/51.97
Margarines and butters	0.00/0.00	2.57/2.72
Cakes and madeleines	0.00/0.00	0.00/0.00
Bricks	0.00/0.00	0.00/0.00
Mayonnaise	0.00/0.00	6.24/10.28

**Table 3:** Data on concentrations of sodium benzoate in marketed foods according to the literature.

Author	Country	Type of Food and Amount of Preservative
Vandevijvere et al., 2009 [18].	Belgium	Carbonated drinks: 128.3 mg/kg. Lemonade: 128.3 mg/kg. Mayonnaise: 585.6 mg/kg.
Lino and Pena, 2010 [19].	Portugal	Carbonated drinks: 148 mg/kg
Soubra et al., 2007 [14].	Lebanon	Carbonated drinks: 120 mg/kg. Juices: 245 mg/kg.
Bemrah et al., 2008 [21].	France	Carbonated drinks: 71.2 mg/kg.
Cressey and Jones, 2009 [22].	New Zealand	Carbonated drinks: 63.1 mg/kg.
Mischek and Krapfenbauer-Cermak, 2012 [23].	Austria	Carbonated drinks: 69.8 mg/kg. Juices: 210.7 mg/kg.
Chaleshtori et al., 2018 [16].	Iran	Carbonated drinks: 61.75 mg/kg. Mayonnaise: 243.42 mg/kg.
Tfouni and Toledo, 2002 [24].	Brazil	Carbonated drinks: 259 mg/kg. Juices: 495 mg/kg.
Azuma et al., 2020 [25].	Ghana	Carbonated drinks: From 5.1 to 277 mg/L. Juices: From 25.80 to 245.10 mg/L.
Magomya et al., 2020 [26].	Nigeria	Carbonated drinks: From 14.25 to 218.32 mg/L.
Shoeb et al., 2022 [27].	Bangladesh	Carbonated drinks: From 96.05 to 285.51 mg/L.
Refat et al., 2022 [28].	Bangladesh	Energy drinks: From 52.92 to 1575.37 mg/L.
Amirpour et al., 2015 [2].	Iran	Juices: 213.2 mg/kg.
Akbari-Adergani et al., 2018 [29].	Iran	Juices: From 12.23 to 56.80 mg/kg.
Yazdanfar et al., 2023 [3].	Iran (Ourmia)	Mayonnaise: From 10.3 to 673 mg/kg.

**Table 4:** Data on concentrations of potassium sorbate in marketed foods according to the literature.

Author	Country	Type of Food and Amount of Preservative
FSANZ, 2015 [20].	Australia	Carbonated drinks: 119 mg/kg. Juices: 191 mg/kg. Melted cheese: 1506 mg/kg. Margarine: 421 mg/kg.
Cressey and Jones, 2009 [22].	New Zealand	Melted cheese: 368 mg/kg. Juices: 216 mg/kg. Margarine: 414 mg/kg.
Leth et al., 2010 [15].	Denmark	Melted cheese: 213 mg/kg.
Tfouni and Toledo, 2002 [24].	Brazil	Melted cheese: 629 mg/kg. Margarine: 532 mg/kg. Yogurt: 183.2 mg/kg.
Amirpour et al., 2015 [2].	Iran	Juices: From 113.8 to 233.3 mg/kg. Yogurt: 36.2 mg/kg.
Magomya et al., 2020 [26].	Nigeria	Juices: From 1.36 to 122.50 mg/L.
Mischek and Krapfenbauer-Cermak, 2012 [23].	Austria	Mayonnaise: 1008.8 mg/kg. Ketchup: 486 mg/kg.
Yazdanfar et al., 2023 [3].	Iran (Ourmia)	Mayonnaise: From 0.005 to 460.3 mg/kg.
Karatasli et al., 2016 [30].	Turkey	Ketchup: 227.27 mg/kg. Mayonnaise: From 0.00 to 892.46 mg/kg.
Chaleshtori et al., 2018 [16].	Iran	Cakes: 127.93 mg/kg.
Mustafa et al., 2021 [31].	Pakistan	Cakes: From 49 to 895 mg/kg.

## Data Availability

The original contributions presented in this study are included in the article/Supplementary Materials. Further inquiries can be directed to the corresponding author.

## Supplementary Materials

The following supporting information can be downloaded at: https://www.mdpi.com/article/10.3390/foods13233880/s1, Table S1: Final sample distribution according to strata and age groups. Table S2: Information about the parents of the surveyed population. Table S3: Estimated dietary exposure of consumers (children and adolescents) in the Oran region to sodium benzoate and potassium sorbate (mg/kg b.w./d) according to living standards. Figure S1: Estimated risk of consumers (children and adolescents) in the Oran region to sodium benzoate. (a): Scenario 1, (b): Scenario 2, (c): Scenario 3. Figure S2: Estimated risk of consumers (children and adolescents) in the Oran region to potassium sorbate. (a): Scenario 1, (b): Scenario 2, (c): Scenario 3.
