# Supplementary material for: Estimation of Dietary Exposure to Sodium Benzoate (E211) and Potassium Sorbate (E202) of Children and Adolescents in the Oran Region, Algeria
Source: Foods. 2024 Nov 30;13(23):3880. doi: 10.3390/foods13233880 (PMC11640519; doi:10.3390/foods13233880)
Supplement: Supplementary file 1 [file foods-13-03880-s001.zip › foods-3270623-supplementary.pdf]

Supplementary file, Table S1. Final sample distribution according to strata and age groups.

Strata	6-10 years		11-14 years		15-18 years		Total
	Girls	Boys	Girls	Boys	Girls	Boys	
Stratum 1	54	58	77	59	97	75	420
Stratum 2	33	24	57	59	86	50	309
Stratum 3	34	16	41	44	32	25	192
Stratum 4	18	13	15	20	16	12	94
Total	139	111	190	182	231	162	1015
	250		372		393		

Stratum 1: municipality of Oran

Stratum 2: municipalities of Hassi bounif, Arzew, Es-Sénia, Sidi Chami and Bir El djir.

Stratum 3: municipalities of Gdyel, Ain Turk, Ain biya, Misserghin, El Karma, Boutlelis, Benfreha, Oued Tlelat, Bethioua, Bousfer and Mers El Kebir.

Stratum 4: municipalities of El Braya, Ain Kerma, Sidi Ben Yabka, El Ancer, Tafraoui, Boufatis, Hassi Mefsoukh, Marsat El Hadjadj and Hassi ben okba.

Supplementary file, Table S2. Information about the parents of the surveyed population.

Parents	Situation	Living standards Low (%)	Medium (%)	High (%)
Father presence	Present	89.58	99.38	99.58
	Absent	2.84	0.31	0.42
	Deceased	7.58	0.31	0
	Total	100	100	100
Father's level of education	Illiterate	11.37	4.68	0.42
	Literate	21.16	10.93	3.82
	Primary school	23.28	12.5	4.68
	Secondary school	25.13	21.87	17.02
	High school	13.75	23.43	27.23
	University	5.29	26.56	46.80
	Total	100	100	100
Father's professional situation	Active	65.60	83.48	85.95
	Unemployed	14.81	2.18	0
	Retired	19.57	14.33	14.04
	Total	100	100	100
Father's profession	Management, decision-making, coaching and guidance	0	4.10	16.83
	Intellectual and scientific professions	1.61	13.80	21.78
	Intermediate professions	0.80	3.35	4.45
	Administrative employees	14.17	17.91	14.35
	Service staff and store and market vendors	7.69	19.40	37.62
	Farmers and skilled workers in agriculture and fisheries	4.85	0.74	0
	Craftsmen	22.26	18.28	3.96
	Installation and machine operators and assembly workers	8.90	11.56	0.49
	Simple and unskilled workers	39.67	10.82	0.49
	Total	100	100	100
Mother's presence	Present	99.30	99.70	100
	Absent	0.23	0.30	0
	Deceased	0.47	0	0
	Total	100	100	100
Mother's level of education	Illiterate	22.19	10.52	1.69
	Literate	17.66	9.59	5.51
	Primary school	14.55	8.97	5.51
	Secondary school	26.01	24.76	19.92
	High school	16.46	30.34	30.93
	University	3.10	15.78	36.44
	Total	100	100	100
Mother's professional situation	Active	6.92	24.76	49.57
	Housewife	92.36	73.99	45.76
	Retired	0.71	1.23	4.66
	Total	100	100	100

Mother's profession	Management, decision-making, coaching and guidance	0	0	5.98
	Intellectual and scientific professions	0	24.05	40.17
	Intermediate professions	3.44	22.78	15.38
	Administrative employees	27.58	26.58	13.67
	Service staff and store and market vendors	17.24	6.32	11.11
	Farmers and skilled workers in agriculture and fisheries	0	0	0
	Craftswomen	10.34	15.18	13.67
	Installation and machine operators and assembly workers	0	0	0
	Simple and unskilled workers	41.37	5.06	0
	Total	100	100	100

Supplementary file, Table S3. Estimated dietary exposure of consumers (children and adolescents) in the Oran region to sodium benzoate and potassium sorbate (mg/Kg b.w./d) according to living standards.

		**Sodium benzoate de (ADI=5)**				**Potassium sorbate (ADI=11)**			
	**Living standards**	**Scenario 2**		**Scenario 3**		**Scenario 2**		**Scenario 3**	
		**Ave**± SE **(risk %)**	**P95 (risk %)**	**Ave** ± SE **(risk %)**	**P95 (risk %)**	**Ave** ± SE **(risk %)**	**P95 (risk %)**	**Ave** ± SE **(risk %)**	**P95 (risk %)**
Surveyed population	Low	0.74±0.03a (14.80)	2.12 (42.40)	0.64±0.03a (12.80)	2.22 (44.40)	2.24±0.07a (20.36)	5.33 (48.45)	1.51±0.06a (13.73)	4.12 (37.45)
	Medium	1.53±0.07b (30.60)	4.16 (83.20)	1.17±0.05b (23.40)	3.57 (71.40)	4.30±0.17b (39.09)	9.83 (89.36)	2.85±0.12b (25.91)	7.26 (66.00)
	High	2.98±0.15c (59.60)	7.96 (159.20)	2.73±0.18c (54.60)	7.37 (147.40)	6.90±0.29c (62.73)	15.49 (140.82)	3.97±0.18c (36.09)	10.02 (91.09)
Children (6-10)	Low	0.66±0.06a (13.20)	2.1 (42)	0.63±0.07a (12.60)	2.27 (45.40)	2.39±0.14a (21.73)	5.07 (46.09)	1.65±0.11a (15.00)	4.13 (37.55)
	Medium	1.61±0.14b (32.20)	4.41 (88.20)	1.18±0.12b (23.60)	3.61 (72.20)	5.88±0.35b (53.45)	13.37 (121.55)	4.02±0.25b (36.55)	9.34 (84.91)
	High	3.61±0.33c (72.20)	7.75 (155.00)	3.20±0.34c (64.00)	6.54 (130.80)	10.45±0.67c (95.00)	18.43 (167.55)	5.98±0.51c (54.36)	12.22 (111.09)
Adolescents (11-14)	Low	0.76±0.05a (15.20)	2.29 (45.80)	0.66±0.05a (13.20)	2.32 (46.40)	2.56±0.13a (23.27)	6.53 (59.36)	1.70±0.11a (15.45)	5.19 (47.18)
	Medium	1.42±0.12b (28.40)	4.01 (80.20)	1.10±0.10b (22.00)	3.55 (71.00)	4.01±0.27b (36.45)	9.57 (87.00)	2.56±0.18b (23.27)	6.93 (63.00)
	High	3.21±0.30c (64.20)	9.68 (193.60)	2.78±0.33c (55.60)	8.61 (172.20)	7.13±0.51c (64.82)	16.69 (151.73)	4.05±0.27c (36.82)	9.58 (87.09)
Adolescents (15-18)	Low	0.76±0.05a (15.20)	1.81 (36.20)	0.62±0.06a (12.40)	1.68 (33.60)	1.78±0.09a (16.18)	4.03 (36.64)	1.20±0.07a (10.91)	2.97 (27.00)
	Medium	1.56±0.11b (31.20)	4.16 (83.20)	1.21±0.08b (24.20)	3.38 (67.60)	3.20±0.19b (29.09)	7.98 (72.55)	2.11±0.14b (19.18)	5.88 (53.45)
	High	2.63±0.19c (52.60)	6.14 (122.80)	2.52±0.25c (50.40)	6.33 (126.60)	5.60±0.35c (50.91)	12.93 (117.55)	3.26±0.24c (29.64)	7.87 (71.55)

Ave: average, P95: exposure to 95th percentile, SE: Standard Error
Same lowercase letters involve no significant differences between both sexes. Same uppercase letters involve no significant differences between the three age groups.

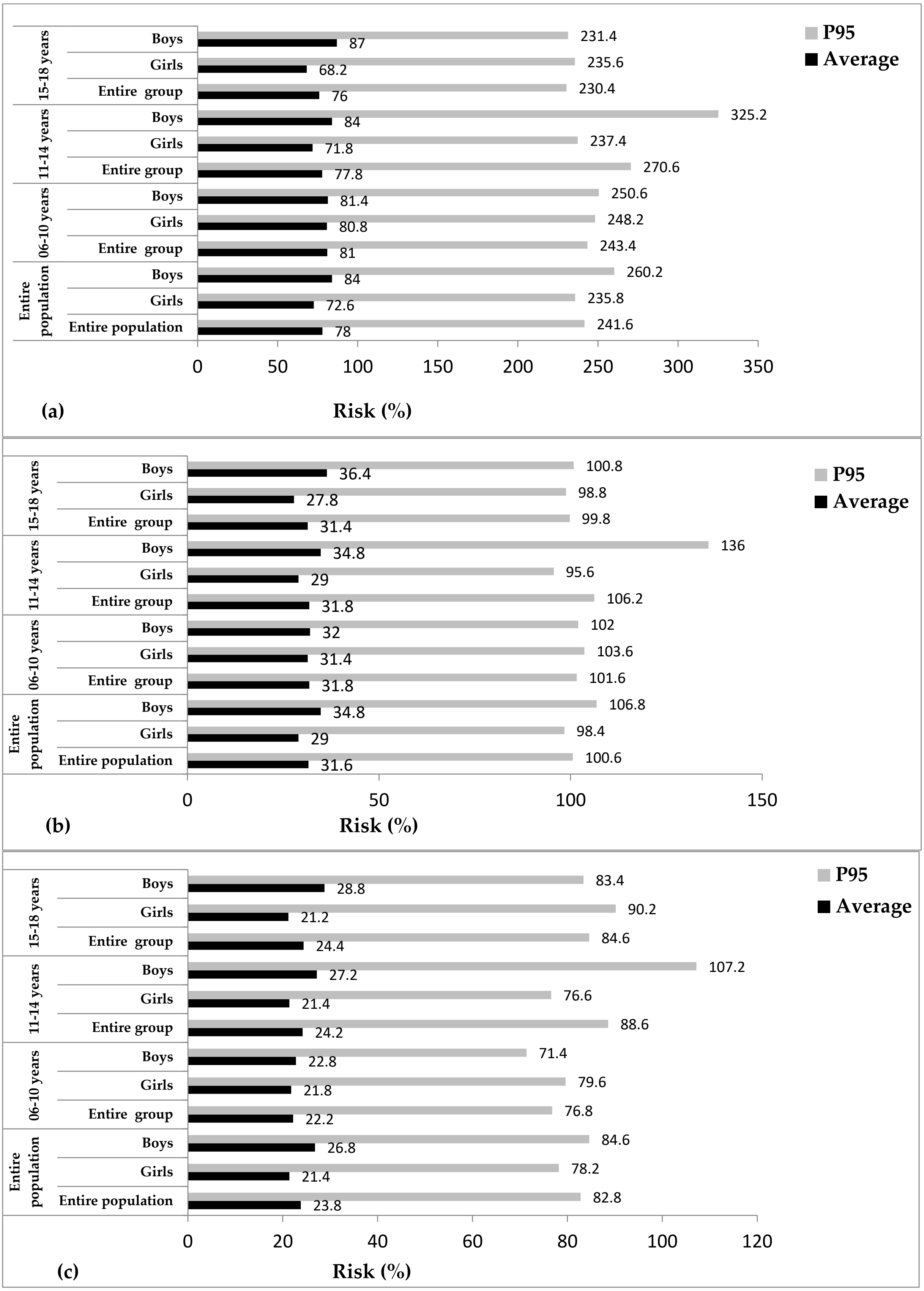

Supplementary file, Figure S1: Estimated risk of consumers (children and adolescents) in the Oran region to sodium benzoate.
(a): Scenario 1, (b): Scenario 2, (c): Scenario 3

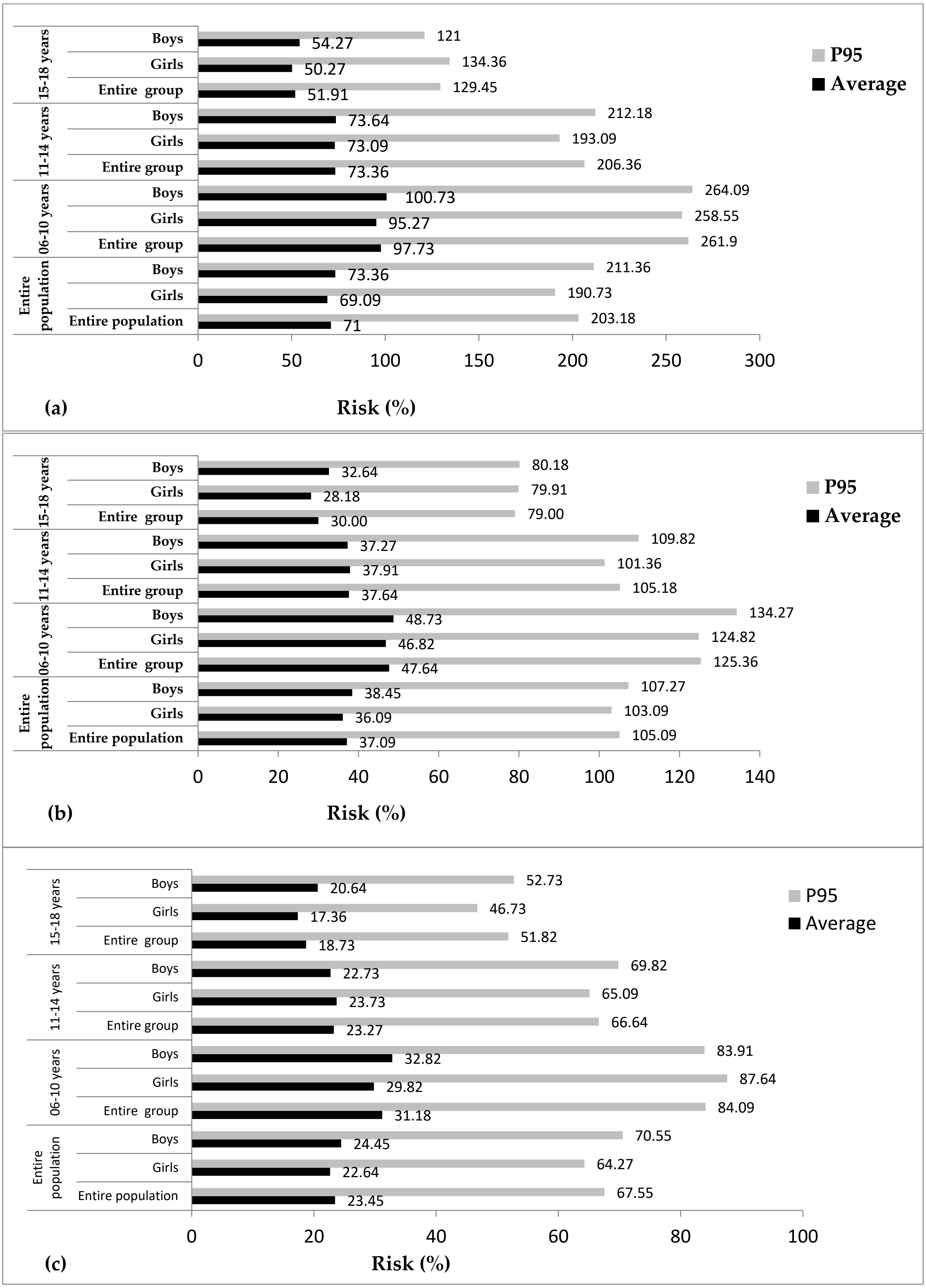

Supplementary file, Figure S2: Estimated risk of consumers (children and adolescents) in the Oran region to potassium sorbate.
(a): Scenario 1, (b): Scenario 2, (c): Scenario 3
